# Yoga Helps Put the Pieces Back Together: A Qualitative Exploration of a Community-Based Yoga Program for Cancer Survivors

**DOI:** 10.1155/2016/1832515

**Published:** 2016-11-16

**Authors:** Michael J. Mackenzie, Amanda J. Wurz, Yayoi Yamauchi, Lanie Ann Pires, S. Nicole Culos-Reed

**Affiliations:** ^1^Department of Behavioral Health & Nutrition, University of Delaware, Newark, DE, USA; ^2^Department of Human Development & Family Studies, University of Delaware, Newark, DE, USA; ^3^School of Human Kinetics, Faculty of Health Sciences, University of Ottawa, Ottawa, ON, Canada; ^4^Graduate School of Sport Sciences, Waseda University, Tokyo, Japan; ^5^Faculty of Kinesiology, University of Calgary, Calgary, AB, Canada; ^6^Department of Oncology, Cumming School of Medicine, University of Calgary, Calgary, AB, Canada; ^7^Department of Psychosocial Resources, Tom Baker Cancer Centre, Alberta Health Services, Calgary, AB, Canada

## Abstract

*Objective*. A qualitative research methods approach was used to explore the experiences of participants in an ongoing community-based yoga program developed for cancer survivors and their support persons.* Methods*. 25 participants took part in a series of semistructured focus groups following a seven-week yoga program and at three- and six-month follow-ups. Focus groups were transcribed verbatim and analyzed using a process of inductive thematic analysis.* Results*. The group was comprised of 20 cancer survivors, who were diagnosed on average 25.40 (20.85) months earlier, and five support persons. Participants had completed the yoga program an average of 3.35 (3.66) times previously and attended approximately 1.64 (0.70) of three possible focus groups. Four key themes were identified: (1) safety and shared understanding; (2) cancer-specific yoga instruction; (3) benefits of yoga participation; (4) mechanisms of yoga practice.* Conclusions*. Qualitative research provides unique and in-depth insight into the yoga experience. Specifically, cancer survivors and support persons participating in a community-based yoga program discussed their experiences of change over time and were acutely aware of the beneficial effects of yoga on their physical, psychological, and social well-being. Further, participants were able to articulate the mechanisms they perceived as underpinning the relationship between yoga and improved well-being as they developed their yoga practice.

## 1. Introduction

Approximately 1.9 million individuals are diagnosed with cancer each year in North America [1]. Medical advances have resulted in improved survival rates for most cancers [2]; however, survivorship is not without its challenges. Cancer is amongst the leading cause of morbidity and mortality in North America [3], negatively impacting psychosocial (e.g., mood and lack of perceived social support) and physical health (e.g., increased risk for comorbid conditions and secondary malignancies; [4]). Given the severity and duration of negative effects, many cancer survivors seek self-management strategies to regain control over their body, lessen the impact of cancer and its treatments on their psychosocial and physical health, and improve their overall quality of life [5]. Yoga has emerged as one such self-management strategy.

Contemporary yoga practice is a gentle activity that combines physical movement (i.e.,* asana*), breathing techniques (i.e.,* pranayama*), and meditation (i.e.,* dhyana*; [6]). In studies with cancer survivors, positive effects have been noted for a variety of outcomes, including mood, cancer-related distress, symptoms of fatigue, and overall quality of life [6–13], though there is some variance in the magnitude of improvements reported [14]. Notwithstanding these discrepancies, the cumulative evidence suggests yoga may be particularly beneficial to combat negative cancer-related effects, holding immense potential as a valuable addition to currently accepted supportive and complementary healthcare services.

Given the preliminary support for yoga in cancer survivorship, further research is required to better understand the experiences cancer survivors have after engaging in yoga programs. Qualitative research is one way researchers may be able to better understand the complex phenomena of yoga participation. Specifically, qualitative research offers distinct advantages by including the voices of participants and providing rich and detailed information that may not be obtained otherwise [15]. As such, a qualitative research approach may be an ideal way for researchers to better understand the complex phenomena of yoga practice for individuals diagnosed with cancer.

To date, few researchers have qualitatively explored cancer survivors' experiences after participating in yoga interventions and programs. van Uden-Kraan et al. [16] conducted a preliminary qualitative exploration with breast cancer survivors participating in weekly yoga classes for 16.8 months. The authors found breast cancer survivors' ongoing participation stemmed from an interest in fostering body awareness, developing relaxation skills, and increasing physical activity as a means to cope with cancer and its treatments as part of rehabilitation. With regard to the self-perceived effects of yoga, participants reported a sense of shared understanding with fellow breast cancer survivor participants and reported experiencing heightened physical fitness, mental strength, resilience, and enhanced coping. Similarly, Galantino et al. [17] explored the benefits of an eight-week yoga intervention for breast cancer survivors coping with aromatase inhibitor-associated arthralgias. The authors found participants experienced an increased sense of camaraderie and community within the program, self-reported pain and stress relief, increased fitness, and an ability to use the skills fostered in yoga practice in daily living (particularly the breathing techniques). Duncan [18] conducted a six-month community-based yoga intervention with a heterogeneous group of cancer survivors; quantitative and qualitative data were collected. Participants self-reported clinically significant reductions in symptoms and mood disturbance and improved quality of life and spiritual wellbeing. The qualitative exploration confirmed and extended these findings, indicating participants attributed improvements in physical well-being to yoga practice and experienced a greater sense of emotional well-being. Moreover, study participants were appreciative of the individualized instruction they received, which they felt bolstered their own self-efficacy (i.e., confidence; [18]). Research conducted by McCall et al. [19] in an outpatient setting found yoga practice reduced stress and other symptoms associated with cancer treatment. In addition, group yoga practice was found to promote prosocial behavior and encourage personal empowerment during cancer treatment and recovery. Taken together, these studies highlight some of the factors underpinning participation in yoga by cancer survivors.

Although these studies contribute to the field, much remains unknown about how participants experience change in yoga practice over time and how self-perceived benefits develop in the context of a community-based yoga program. Further, perspectives from support persons have yet to be explored. Including the voices of support persons' experiences in a yoga program are necessary as cancer's impact extends beyond the patient. Support persons also report impaired physical, psychological, emotional, and social functioning and well-being, resulting in increased levels of distress and reduced quality of life [20]. Thus, research is required to provide an in-depth understanding of both cancer survivors and their support persons' participation in a community-based yoga program. The present study sought to address this gap by exploring both cancer survivors and support persons experiences following a seven-week community-based yoga program. A qualitative research approach was used to explore participants' experience of change over time and better understand how self-perceived benefits are accrued via the phenomenological lens of the participants in the yoga program.

## 2. Methods

### 2.1. Participants

Ethical approval was obtained from a Research Ethics Board (REB) and all participants completed an REB-approved informed consent prior to study enrollment. Program participants were comprised of a heterogeneous group of cancer survivors enrolled in the ongoing “Yoga Thrive: Therapeutic Yoga for Cancer Survivors” program, as described previously [21]. The Yoga Thrive program is a research-based, therapeutic yoga program for cancer survivors and their support persons. This seven-week program is based on contemporary yoga practices modified for cancer survivors [21, 22]. Participants were eligible for study inclusion if they (1) were aged 18 years or older and (2) had received a cancer diagnosis at any time previously or (3) were a support person of a cancer survivor coattending the Yoga Thrive program. Previous participation in the Yoga Thrive program was not an exclusion criterion but was evaluated as part of the study. Participants were informed of the research study at the time of registration for the Yoga Thrive program, via either telephone or email. The study coordinator then contacted those who indicated interest in participating in the qualitative component of the research study.

### 2.2. Procedures

Once recruited into the qualitative portion of the study each participant engaged in up to three separate one-hour semistructured focus groups completed after the program (i.e., following the seven-week yoga program or at three- and six-month follow-ups). The use of several focus groups allowed participants to describe how their experience of yoga practice shifted over time. In total, ten focus groups were conducted over a period of six months (four at follow-up, four at three-month follow-up, and two at six-month follow-up). Focus groups were comprised of both cancer survivors and their support persons who had participated in the Yoga Thrive program. The aim of the focus groups was to elicit conversations concerning participants' experiences in the yoga program in conjunction with their cancer experience (see Qualitative Research Questions). The focus group approach permitted discussion and allowed for data to enter the interview that was not directly sought, thus allowing participants to provide information they believed was important and relevant to them [23]. As a result, questions asked varied between each focus group. Main points were summarized by the researcher at the end of each focus group for the purpose of member-checking and credibility [18]. All focus groups were conducted in the community and audiotaped with consent.

*Qualitative Research Questions*

*(1) Post-Seven-Week Yoga Program Focus Groups*

- Tell me about your experience in the yoga program.
- Why did you decide to participate in the yoga program?
- What did you expect when you started to practice yoga?
- What was it like for you when you started out?
- What has changed for you practicing yoga from the beginning of the program until now?
- What motivated you to continue the program?
- What do you think you have learned from this program?
- What did you like best/least about this program?
- What do you think are some of the benefits/deficits to practicing yoga?
- What effects, if any, have you noticed since joining the yoga program?
- What role does yoga play in your cancer recovery?
- When people don't practice yoga as often as they'd like, why not?
- What suggestions do you have for us to improve the program?
- What additional information would you like to add today?

*(2) Three- and Six-Month Follow-Up Focus Groups*

- Tell me about your yoga practice since completion of the program.
- What has changed for you practicing yoga from completion of the program until now?
- What effects, if any, have you noticed since completing the yoga program?
- What has motivated you to continue practicing yoga?
- What do you think are some of the benefits/deficits to practicing yoga?
- What role does yoga play in your cancer recovery?
- When people don't practice yoga as often as they'd like, why not?
- What additional information would you like to add today?

### 2.3. Qualitative Analysis

All focus groups were transcribed verbatim and analyzed using the NVivo 9 computer program (QSR International, 2010). The program allows for the organization of textual data into categories, themes, and subthemes and facilitates management of a large volume of textual data. All focus groups were conducted by a single researcher (MM) then coded independently by two research assistants (AW and YY) using a process of inductive thematic analysis [15]. Inductive thematic analysis is a process of coding that allows the data to drive the analysis (as opposed to deductive analysis which involves fitting the data into preexisting categories or themes). Both coders reviewed the transcripts several times to familiarize themselves with the textual data and then raw data quotes were identified, labeled, and organized into key themes. Common themes were then combined into higher order categories. These preliminary themes and categories were compared and contrasted between the research assistants to assure consistency of coding and to ensure all data were accounted for by core categories. Emerging themes were triangulated with the inclusion of several participant cases [24]. Codes identified by the two research assistants were then reviewed and verified as a team with the principle investigator and a second coder (MM and LP) over the course of several meetings. Next, the thematically categorized research findings were integrated with the subjectively reported experience of participants. Specifically, direct quotes were provided from participants to substantiate key themes and ensure transferability of categorical assertions made by the researchers, so that readers could experience participant perspectives in their own words [18].

## 3. Results

### 3.1. Participants

The group was comprised of 20 cancer survivors (*M*_age_ = 56.14; SD = 8.93) who were diagnosed on average 25.40 (20.85) months earlier and 5 support persons (*M*_age_ = 71.73; SD = 18.88). Participants were predominantly female (88%) and had completed treatment for breast cancer (*n* = 11), cervical cancer (*n* = 1), colorectal cancer (*n* = 2), lymphoma (*n* = 2), ovarian cancer (*n* = 2), and prostate cancer (*n* = 1). Treatment modalities included surgery (*n* = 16), chemotherapy (*n* = 13), and radiation (*n* = 11) and/or combinations thereof. Participants had completed the Yoga Thrive program an average of 3.35 (3.66) times previously and attended approximately 1.64 (0.70) of three possible focus groups.

### 3.2. Inductive Thematic Analysis

Qualitative analyses of the focus groups offered insight into participants' experiences with the yoga program. Four broad themes were identified: (1) safety and shared understanding; (2) cancer-specific yoga instruction; (3) benefits of yoga participation; (4) mechanisms of yoga practice.

#### 3.2.1. Theme 1: Safety and Shared Understanding

Participants indicated attending a yoga class comprised of other cancer survivors provided feelings of safety and shared understanding of the cancer experience: “It's a very safe environment. We all have that common thread (cancer). It kind of draws you together and helps you to open up.” Shared understanding of cancer was a uniting force for participants providing feelings of comfort and support: “Whether you are the spouse or partner of someone who had cancer, or actually had cancer yourself, everybody is kind of healing. It starts to make you a survivor instead of a victim of it (cancer).” Despite being at different stages (i.e., on-treatment, off-treatment, and support person), participants still felt connected to one another: “You are all at different stages physically and different stages in recovery, but you have all been through it (cancer) or are going through it, so that means a lot.” This sentiment was common: “Coming here with other cancer patients, I guess we're all in the same kind of… gone through similar experiences.” This identification with other cancer survivors though oft-repeated was summarized when a participant reflected on her experience when a new cancer survivor joined a yoga class she was registered in: “When she walked across the room I thought,* ‘wow, that's all of us…'* That's why I think you feel comfortable here.” Participants attributed this shared meaning through the cancer experience as a safe place in which they could work together to learn a new skill: yoga.

For several participants the safety and shared understanding within the cancer-specific yoga program precluded them from trying yoga elsewhere, “I thought,* ‘I will do yoga elsewhere*.*'* Well that was the biggest mistake… I could not keep up and I walked out of there almost in tears. I felt so horrible, thinking I was doing so well, then I go to a class and can't do it. It was a heart wrenching experience… I hate to say it, I am almost afraid to step outside that boundary (of Yoga Thrive) because I don't know what to expect.” Another participant shared, “I did sign up for yoga in my community at the same time. I did the Yoga Thrive and I thought,* ‘I'm not doing the community one.'* It was just too scary. For me, I wasn't comfortable telling people that I had cancer. I don't want to go into another class and tell them,* ‘Well I have had cancer and I have these injuries.'* I didn't want that. I didn't feel that with (Yoga Thrive). It was like everybody is in the same boat.” For another participant who had suspended her regular yoga practice during treatment, “Yoga was so restorative. It was just wonderful and such a good step to come back into yoga because I had done it previously. I took that whole year (cancer diagnosis and treatment) off, and got away from it (yoga) then I built up this fear of going back into regular mainstream yoga. When I saw that poster (Yoga Thrive) I took it off the wall so I would have it for the minute I was done (cancer treatment) and could get back into it because I loved it before and equally am finding it so beneficial now.”

The discussions around the cancer-specific nature of the yoga program were complex. For many, despite acknowledging the benefits of a shared experience, it was the focus on yoga and not their cancer experience that was valued. “The cancer thing was too overpowering for me. I didn't want to be about that. So (yoga) is not about cancer. I have tried doing other cancer things and I am just not taken to it anymore… I have moved on.” Several other participants commented on the importance of shifting the emphasis from shared cancer experience to yoga practice itself. “It's not focused on the cancer but the focus is on the well-being of the individual and that's very positive.” Another participant shared, “I didn't really want to belong to a* ‘cancer club'* afterwards (post-treatment)… but the yoga appealed to me because it was physical, but then it was just such a safe, nice environment.” Extending this notion, for a small group of participants, it was the focus on improving their own health and well-being, and not necessarily being around similar others that was important. As one participant stated, “I really wanted to find something that was just going to be for me – to help me feel better, to feel stronger. It had nothing to do with cancer. That's what I love about this yoga.” Similarly, another participant said, “It really had more to do with helping me period,* ‘Help me – Me'* which really in my head had nothing to do with cancer at the time.”

While participants appreciated the shared understanding and safety of practicing yoga within a cancer-specific program, many expressed fear around engaging in yoga beyond Yoga Thrive. The camaraderie they found within the cancer-specific yoga program was second for many to the focus on the practice of yoga itself within this environment.

#### 3.2.2. Theme 2: Cancer-Specific Yoga Instruction

In addition to feelings of safety and shared understanding, many participants commented on the cancer-specific yoga training of the instructors and the corresponding individualized and progressive nature of the yoga instruction, “The strength of the program is the way of instruction. It's very compassionate, humane and encourages you to try different things and continually grow. It's sort of the opposite of* ‘no pain no gain'*… It's very respectful of your body.” Participants suggested that given the nature of class instruction this further engendered a sense of safety and ability to work to one's fullest potential throughout each class.

*One of the neat things during the class is that I have never felt intimidated by not being able to do something. I love the fact that there is always a suggestion (from the yoga teacher), “Well if you can't do that you can try this instead, and, you're still getting some benefit from it.” You're not sitting there waiting for everybody to finish the pose, so you're always a part of it, you're participating in it.*

This theme was reiterated among several participants.

*I am going to attend a class instructed by somebody who understands I may have limitations because of my physical experience - whether it be surgery, chemo, radiation or whatever else… I wanted to be in a class led by somebody who would… not just respect I have limitations, but also understand what they might be, and know what I should and shouldn't do.*

Participants appreciated the yoga instructors had received cancer-specific training, even if they did not recognize it consciously when they first registered for the program. “So yes, it's somebody who would understand and not just respect that I have limitations initially, but also understand what they might be. That would obviously have to be somebody who would understand that if you had a mastectomy if you had lymph nodes removed or if you didn't and therefore, what restrictions you may have physically and what you shouldn't be doing as well. Not just what may not be able to but what you shouldn't be doing. So now that - that was probably subconsciously.” This specialized instruction was a key factor in putting participants at ease, largely informing their decision to participate in yoga. However, it should also be noted that for some this appreciation for cancer-specific yoga training stemmed from fear of injury and the role of the instructor in potentially mitigating perceived harm: “I did it (cancer-specific yoga program) because I was afraid of injuring myself. I thought if I went to any other yoga they wouldn't know (understand) what had happened. I felt this would cater more to people who had surgery or were going through other treatments… I wasn't afraid of going to this.” Given the varied levels of practice and physical and psychosocial health that participants entered the program with, the adaptations the instructors offered were greatly appreciated. Whether specific to their cancer experience or not, participants suggested the challenge of yoga practice and trying the different yoga postures allowed them to, “find out where my body wasn't quite doing it the way I should be, and knowing where I needed to work on…There's a bit of challenge to think about each time and what could I work on to get a little better at.” Another participant stated, “There was a pose variation where you could put your foot against your thigh or higher on your ankle. All I could manage was the ankle. I appreciated that adaptability to the range of abilities that were there.”

Interestingly, for this study, participants had a wide range of experience with the Yoga Thrive program. For those who had taken the program before versus first time participants, experienced participants appreciated ongoing modifications the yoga instructors provided as they continued to practice. “There are different levels: some are new and some have been there a long time. She (the yoga teacher) is able to give us a little bit more, for those of us who have been there for a while. Just a bit more information about the pose, minor details that you wouldn't introduce at the beginning but now we are ready for. So now you can focus on it. You are learning and doing more that the last time you did the class… Even though we have done it so many times, I still find I learn something every time.”

Participants appreciated the yoga instructors' cancer-specific training. They shared that this also provided a degree of safety and shared understanding, allowing instructors to offer appropriate modifications for participants based on their current needs and abilities. For more seasoned practitioners more detailed instruction allowed them to further grow over time as they improved and deepened their yoga practice. Although classes were comprised of individuals at varying levels on the cancer trajectory and a range of levels of yoga experience, the ability of the yoga instructor to adapt, modify, and safely guide the group was helpful across the cancer and yoga experience continuum.

#### 3.2.3. Theme 3: Benefits of Yoga Participation

Although cancer and its treatments exert a tremendous toll on cancer survivors and support persons, in the program, yoga was described as both a respite and means of taking control back: “For me it was very restorative and helpful for me to start as soon as I did.” Support persons agreed with this sentiment and also found yoga to be a reprieve in the ongoing support they provide.

*What I think I got was you come with your own burden as a family member around the responsibility and what it looks like in the future and what's that going to mean. The benefit I didn't appreciate before. I was thinking I was helping just by signing us up. I think it relieves your own stress by normalizing some of that discussion around cancer if it came up. People who were living with it in different ways, seeing other people cope with it. I found it relieved my own stress around being the support system for someone who was living with cancer. That was an added benefit. I thought I was helping him (by co-attending the program), but it really does help as a partner in that sort of progression of living with cancer. I didn't anticipate that. That's why I enjoy it too, because it relieves some stress and takes some of that burden away to feel like I am the only option… it has provided in the same way for me as it has for him. I wasn't expecting that.*

This feeling of relief from the symptoms of cancer and its treatments was attributed in part to the control participants felt the yoga program gave them: “I like to feel I have some control over my life. That is one of the huge benefits I have got from the program…” Several participants echoed this sentiment.

*You're never discouraged – it's something I have chosen to do: something I have control over. It is at a time in your life when you have very little control over anything else that could happen physically to you. So that to me was huge. I make the decision, “I am attending. I can do this.” It was getting that control back of my body I guess really… I don't know how you can get that across to people?*

In addition, participants used the yoga program to confer psychosocial and physical benefits. Specifically, the most commonly reported benefits of yoga were an increased ability to relax, concentrate, and regulate emotions, manage symptoms of fatigue, pain, and sleep difficulties, and promote physical health. For example, participants commented on how yoga allowed them a space to relax and encouraged them to slow down. One participant commented: “This is more gentle and relaxing - it's just a different way of doing things…I think the yoga just kind of calms your whole body and mind.” Another participant further suggested yoga, “helps me go into my body or heart instead of my mind. It slows it all down.” Yoga also honed participants' ability to concentrate: “To stop and focus,” and regulate their emotions. The emotional benefits often occurred acutely, with participants noticing a significant shift before to after class: “I leave in a good mood every single time. I have never left yoga in a cranky mood ever.” Yoga's utility to assist with symptom management was described by many participants. Other respondents described how yoga improved their physical health by improving their mobility and strength: “It really does help physically…I could feel my physical strength improve.”

For many there was also a shift in focus from the physical to mental benefits of yoga. “I think I originally enrolled for the physical benefits. The mental benefits came later.” One participant stated, “It took me, I would say, the majority of the time to just calm down and take in more of the therapeutic aspect and enjoy it. At the beginning I just kept thinking,* ‘OMG this isn't getting me anywhere'* and I couldn't focus. I just wanted to get more physical, thinking that's what I needed…At the end I thought,* ‘Wow that was good!'* I just calmed down and my whole perspective changed. I started enjoying the class.” These benefits were often also experienced outside of class, “When I am worried or thinking or going off, I will stop and just try and breathe. I try to incorporate what I have learnt in the class.” Others posited emotional benefits, “If I am feeling emotional or some stress in my life before I would just keep spinning and spinning and getting tighter and tighter. Now it's like I have learned a tool to help me relax and let go of a lot of stuff. It's tremendously emotionally helpful. The techniques, tools you learn in yoga are applicable to everyday life: everywhere you go, everything you do.” Another participant suggested, “One thing it has done for me is help me separate myself from situations so that my emotions and moods don't get tangled up in something that doesn't have to do with me personally, it's just the situation I happen to be in. It helps me take one step back and look at it from an outside perspective. Put things in place where they need to be.”

Although many spoke of the benefits of yoga practice, these changes were not immediate for many, “The first while doing down dog (a yoga posture) was killer but I just… did it and now I shock myself at my strength in some of that stuff… The chemo had tightened all the muscles in my body and so I think going to yoga has helped counteract that.” Another shared, “I remember first starting out and I wasn't very good at anything, never mind getting on the floor, but when the instructor would say,* ‘You got it!'*… It was almost like,* ‘Wow, I was never able to do that and now I can do this!'* Those little moments make you feel really good because you've come a really long way in comparison to what you were doing. I'm still struggling, but it's like a goal to reach for. I may never be good at all of it, but it's one pose at a time.” Another participant shared, “First of all I didn't really know what to expect… Then when you do the moves, you don't really have the feel of your body… When you start to know what to expect you have that mind-body thing… You start to be calm and can focus on the moves, you can go much farther. I don't know how to explain it… it is like a mind-body thing. You feel your body and you not only see the instructor and everybody else, but you actually start to focus on yourself. I didn't do that in the beginning.”

Although the majority of participants commented on the benefits they received through yoga practice, some participants highlighted that fatigue, a common side effect, was a significant barrier that may have mitigated the full extent of the benefits they received from program participation. “As far as fatigue goes, I don't know if yoga has played a part in it… of the entire experience that's the one thing I struggle with. It's taking such a long time to come back. I can see a huge shift from where I was, but I still have that (fatigue). If I wasn't in yoga would it be any better? Hard to say.” Another participant offered, “The yoga was good even when I did take it when I was tired. I did get some benefit even as I realized at the time that it was kind of a chore to go… Even though it was once a week some nights I thought,* ‘Well I signed up, this is supposed to be good for me…I'm just really tired. I'd rather just stay home.'* But anyways I went.” Participants also clearly and candidly described how the benefits accrued varied based on where they were in their cancer treatment continuum (i.e., on- or off-treatment). Some participants indicated they did not receive the full benefit of yoga practice initially. “I had just finished chemotherapy and radiation when the yoga started. I thought it would be okay but… I think all of the treatments started to accumulate and I was really tired all the time. I didn't get as much benefit out of it as I should have I think. Whether mentally I was tired or physically I was tired, it was just not the right time for me, you know?” Despite the lack of change, the participant continued in the program, “A year later was much better and I really enjoyed it.” This feedback suggests yoga may have had limited utility in combatting more pronounced treatment-related side effects during active treatment. That said these individuals continued their yoga practice.

Finally, participants were careful to fully ascribe their recovery to yoga practice, “I am very careful to say if it is the yoga or that I am getting rid of all the poison I had in me with the chemo. I know I'm going much farther with the yoga now because I'm at peace. I'm ready to do things when I'm finished with my (yoga) exercises.” Another participant commented, “I am back to doing everything, back to where I was prior. It could be the yoga, it could be a bunch of other contributing factors, but I am still doing my yoga with the belief that it helps me. It is good for my continued recovery.”

Participants described a host of physical, mental, and emotional benefits from yoga practice inclusive of symptom management. However, these benefits were not immediate and rather were a function of continued yoga practice. Fatigue and treatment-related barriers were often highlighted as challenges participants faced in their yoga practice and in many cases impeded the full range of benefits from being derived. In general participants did not view yoga as a panacea but saw it as an important component of their cancer recovery.

#### 3.2.4. Theme 4: Mechanisms of Yoga Practice

Many participants offered their own explanation for the benefits they accrued via yoga practice, such as growing awareness of the mind-body connection, improving breath regulation, taking time to stop and slow down, and increasing confidence: “It really is more of a spiritual, emotional, and physical experience. I think that's what the yoga program provides that you aren't going to get anywhere else.” Several participants reported how they noticed improvements in other aspects of their lives as a function of these mechanisms.

*Yoga was the first time I realized I could lower my blood pressure by the way I breathed. I could take a breath and feel the stress drain away. The first time I saw that mind-body connection very clearly and I have used it actually quite often over the last three or four years.*

Many of the participants attributed improved breath regulation to physical and mental benefits.

*I do have some lower back problems and I know I get tense. One thing I have learned to do is to breathe into those spots, which is how your get your muscles to relax. And I learnt that from the yoga program, nowhere else. *

Moreover, yoga improved participants' confidence, in their ability to not only practice yoga, but also engage in activities outside of the studio: “It has given me more confidence to stretch that out a bit in my own home, in another gym, things like that. I never would have joined that gym if I hadn't gone to the yoga program already.” This notion of improved confidence came up several times in many different ways.

*There was more confidence overall in myself. With confidence came an overall strength. It's the can do attitude that starts evolving from that as well. It's like a dominos thing - or a snowball really. It just keeps building and building and building…. *

Another participant agreed: “It was a sense that if I can do this yoga piece then I can do that home piece. If I can get up the stairs at the yoga studio I can get up the stairs at home, or if I am strong enough to do* this*, then I can do* that.*” Through cultivating heightened awareness of the mind-body connection and psychophysiological self-regulation skills, participants developed the benefits of yoga both on and off the mat in activities of daily living.

While these proactive mechanisms by which participants posited the benefits of yoga accrued, it is important to note that, in many cases, participation in the yoga program by cancer survivors was motivated by fear.

“For me, it's all about the mobility. I am really afraid. I still suffer from neuropathy, so I am just really afraid that if I stop I won't be able to move again. It' almost a fear-driven thing for me, besides the fact I've made friends … and I feel good when I do it. I feel energized when I leave. I mean there is lots of benefits, but I think the main reason is that I am always afraid I am not going to be able to move again if I don't continue.”

In addition, another participant stated, “If there is anything I can do to stop any kind of recurrence, for my kind of cancer there is an 80% chance…. So if there is anything remotely, minute that could prevent that (recurrence), I am willing to try it… Maybe it's a fear driven thing? My mother ended up with four different kinds of cancer over her lifetime and I don't want that to happen to me. So maybe it is fear.” Another survivor added, “This yoga helps me get through that because sometimes I am terrified. I am just terrified of what happens if this comes back. I am not afraid of dying. I am afraid of going through this again and it wasn't even that bad. But I don't want to go through this ever again.”

Participants believed yoga practice benefits were derived from heightened awareness of the mind-body connection, breath regulation, better self-regulation, and improved self-confidence. However, in this sample, incentive to practice was also be related to coping with fear of worsening symptoms and cancer recurrence.

## 4. Discussion

Approaches to reduce the burden of cancer and its treatment for the growing population of cancer survivors are necessary. Self-management strategies, such as yoga, hold promise to allow individuals to regain control and manage their psychosocial and physical health [25]. Yoga has become more widely available within the cancer community as part of cancer treatment and recovery [11]. The impetus for the present qualitative study was to further explore cancer survivors and support persons' experiences of change over time and how self-perceived benefits are accrued via the phenomenological lens of the participants in the yoga program. This set of exploratory focus groups identified the following four key thematic areas of interest in a community-based yoga for cancer survivors program, including (1) safety and shared understanding; (2) cancer-specific yoga instruction; (3) benefits of yoga participation; (4) mechanisms of yoga practice.

The first theme, “safety and shared understanding,” highlights the cancer-specific nature of the program provided feelings of safety and shared understanding. Cancer survivors and their support persons make deliberate choices to attend programs where there is a shared sense of meaning and collective understanding via the cancer experience [26]. Cancer-specific programs provide a place for survivors to engage in yoga with others who have also been affected by the disease. Previous literature suggests sharing a similar cancer diagnosis can create a sense of community that may alleviate of a sense of isolation commonly experienced by patients facing cancer [19]. In this study, shared practice conferred additional benefits independent of social support. Many participants iterated their appreciation for being able to engage in yoga within a cancer-specific program, highlighting the centrality of the yoga practice (and not their cancer) as important. Given yoga classes are experiential, with relatively little time devoted to talking and sharing amongst participants [17], a community-based yoga program not only may provide physical benefits, but also may provide an ideal avenue by which participants, who are not inclined to engage in social support groups, can have their social support needs met. This highlights the important role yoga may be able to play for cancer survivors; however, the relationship between the cancer-specific aspects of the yoga class in conjunction with participants' shared experiences and the seemingly contradictory desire to distance oneself from a “cancer support” group is an area worthy of further exploration.

The second theme, “cancer-specific yoga instruction,” suggests cancer survivors and their support persons not only view their cancer experience as unique but are aware and appreciate (1) that the yoga program is cancer-specific, (2) that the yoga instructors had appropriate, cancer-specific training, and (3) that the unique circumstances and symptoms of cancer survivors can be worked through in a collaborative fashion. A previous study conducted with breast cancer survivors found that a cancer-specific physical activity program was perceived as safe and supportive when the instructor was regarded as being trained and experienced in cancer and exercise [27]. However, the skills fostered within yoga practice are cultivated overtime and participants were appreciative of instructors' ability to meet participants where they were at in their cancer recovery and their corresponding levels of physical and mental fitness [28]. This included those new to yoga in need of more immediate modifications based on cancer treatment status or other functional limitations, as well as those participants who had established a yoga practice but were looking for additional instructional depth to facilitate their yoga experience. Moreover, similar to other qualitative work [17], the current findings suggest that the instructor played a central role in many of the benefits accrued (e.g., confidence). This came from learning yoga within a safe and supportive environment comprising positive interactions with instructors and fellow yoga participants.

The third theme, “benefits of yoga participation,” underscores the multitude of benefits participants achieve from engaging in yoga practice, from stress reduction and relaxation to physical health promotion, social-emotional regulation, and improved quality of life. Similar to work by McCall et al. [19] yoga was reported by participants as a means to encourage personal navigation of the cancer experience. Participants (survivors and support persons) indicated that participation in the community-based yoga program was a respite both during and following cancer treatment. Cancer survivors suggested yoga practice leads to a heightened sense of self-awareness and empowerment during a difficult disease and treatment course and in time of increased distress and uncertainty. Moreover, yoga served as a means to better self-regulate and manage symptoms including pain, fatigue, and sleep disturbances. Generally, the psychosocial and physical benefits reported were similar to those reported in recent systematic reviews and meta-analyses [10, 11]. The results therefore extend previous findings that yoga exerts positive health benefits for cancer survivors. However, in addition to confirming some of the psychosocial and physical benefits reported by recent reviews, this study also provides evidence that yoga may promote outcomes that have been studied less frequently, including relaxation and mental health.

Despite the reported benefits, it is important to temper these findings per the focus group discussions. Namely, that these benefits were accrued over time and cancer and its treatment posed significant barriers to attaining these benefits. This has implications for how yoga programs are presented and discussed with cancer survivors. Explaining that change is a process and benefits accrued may vary depending on treatment status may better prepare cancer survivors for participating in a yoga program and for their outcome expectations. Finally, while participants felt yoga practice aided in their cancer recovery, they by no means felt it was a remedy for all their ills. Many participants indicated they were also engaging in a range of other self-management strategies, while others indicated they felt better as they recovered as a function of time since treatment. This could indicate that yoga may be an invitation to other self-management strategies or vice versa. While more studies are needed to evaluate the effects of yoga on these outcomes, this study provides preliminary evidence that yoga may be a useful self-management strategy to promote facets of psychosocial functioning, within the context of survivorship.

The fourth theme, “mechanisms of yoga practice,” suggests cancer survivors and their support persons are acutely aware of the process by which the benefits they accrue from yoga practice are derived. The mechanisms participants reported were similar to those that have been reported previously in studies exploring yoga with cancer survivors (i.e., awareness of the mind-body connection, ability to regulate breath, and enhanced confidence; [21, 29]). However, in addition to these yoga-specific elements, participants also described intrinsic motivations as a contributor to the benefits obtained. This sense of regaining control has been evidenced in other qualitative research [18], suggesting participants may benefit from engaging in self-directed practices, which allow them to take an active role in their cancer recovery. This mirrors research conducted in the broader physical activity literature that has found self-determined motivations to be strongly associated with psychological outcomes [30–32].

Extending work by van Uden-Kraan et al. [16], participants felt their participation in yoga contributed to their own cancer rehabilitation process. However, this mechanism was tempered with participant fears of worsening symptoms and cancer recurrence [33], as this was a strong motivator for beginning and continuing engagement in the yoga program. There is no consensus on the definition of “fear of recurrence,” but many suggest it is the “fear that cancer could return or progress in the same place or in another part of the body [34].” Fear of cancer recurrence is one of the most commonly reported emotional effects of cancer [35], with the majority of studies finding that fear of cancer recurrence is one of the most consistently reported unmet needs [36]. Interventions are only recently being developed and delivered (e.g., counseling, cognitive-behavioural therapy) to target fear of cancer recurrence. A recent systematic review found few trials and mixed results for intervention efficacy [37]. Participants' experiences in this study therefore highlight several important avenues for future researchers, such as examining if yoga mitigates or tempers fears of recurrence and exploring individual differences in fear of recurrence as a motivator to engage in yoga.

### 4.1. Strengths and Limitations

In contrast to previous yoga studies that have focused on breast cancer survivors, this study included mixed cancer survivors and their support persons. The inclusion of a heterogeneous sample ensured multiple perspectives were included. In addition, this study included a relatively large sample size for qualitative research. The use of a qualitative research approach in conjunction with inductive thematic analyses across 10 focus groups over a period of six months provided a unique analyses of how yoga practice in a community setting is perceived by cancer survivors and their support persons. Thus, these findings extend previous research, but also provide novel information identifying potential mechanisms underpinning the benefits accrued, highlighting the complexities of participating in yoga in the context of survivorship, and provide an in-depth understanding of participation in a community-based yoga program. This study lays a foundation in the aid of creating stronger yoga research evidence and heightened clinical practice for those facing cancer.

Despite these distinct advantages, this study is associated with several limitations that should be considered. Inherent within qualitative methodology, findings cannot be generalized beyond this group of informants, who are unique in their diagnoses, backgrounds, and personal histories. Rather qualitative studies of this nature can be used to generate hypotheses rooted in firsthand experiential data of how cancer survivors perceive their experience of yoga and the role it may play in their lives. In addition, the average participant in the qualitative component of this study had taken the program three or more times. This constitutes a unique group of cancer survivors, many of whom had ample yoga experience. Given this familiarity with yoga practice the benefits of practice they describe may not be achievable in those who practice for a shorter duration. Given the heterogeneity of cancer types and duration of practice, a dose-response relationship may also be important to explore [38]. However, these findings are consistent with other qualitative and quantitative research findings in both yoga and physical activity research, which enhance confidence in the suggested benefits and associated mechanisms of action.

## 5. Conclusions

Participants shared their collective experience of yoga practice as cancer survivors and support persons. The present study generated significant contextual knowledge about the role of yoga within cancer survivors lives over the course of several months within a real cancer care setting. In general, the present qualitative findings suggest yoga was an important component of survivorship aiding cancer survivors in their cancer journey and provided respite during active treatment. Participants in the current study experienced yoga practice as beneficial combining tailored methods of self-regulation within a socially supportive safe environment that was focused on yoga practice instead of “cancer.” Further research is needed to further explore these preliminary findings for yoga practice within community-based cancer care settings. It is suggested that continued qualitative research or mixed methods research methodologies be utilized in to better understand and explore participants lived experience of yoga and how these practices become integrated into their lives. Given the diverse benefits and posited mechanisms, more structured research will further identify the beneficial aspects of yoga within a socially supportive environment. These findings may inform future directions of research, providing a rich phenomenological source from which further questions and theories can emerge concerning those who practiced yoga in tandem with mainstream cancer treatments.
